# Composite Bonding to Stainless Steel Crowns Using a New Universal Bonding and Single-Bottle Systems

**DOI:** 10.1155/2013/607405

**Published:** 2013-04-02

**Authors:** Mohammad Ali Hattan, Sharat Chandra Pani, Mohammad AlOmari

**Affiliations:** ^1^Division of Pediatric and Preventive Dentistry, Riyadh Colleges of Dentistry and Pharmacy, P.O. Box 84891, Riyadh 11681, Saudi Arabia; ^2^Division of Restorative Dentistry, Riyadh Colleges of Dentistry and Pharmacy, P.O. Box 84891, Riyadh 11681, Saudi Arabia

## Abstract

*Aim*. The aim of this study is to evaluate the shear bond strength of nanocomposite to stainless steel crowns using a new universal bonding system. *Material and Methods*. Eighty (80) stainless steel crowns (SSCs) were divided into four groups (20 each). Packable nanocomposite was bonded to the lingual surface of the crowns in the following methods: Group A without adhesive (control group), Group B using a new universal adhesive system (Scotchbond Universal Adhesive, 3M ESPE, Seefeld, Germany), and Group C and Group D using two different brands of single-bottle adhesive systems. Shear bond strengths were calculated and the types of failure also were recorded. *Results*. The shear strength of Group B was significantly greater than that of other groups. No significant differences were found between the shear bond strengths of Groups C and D. The control group had significantly lower shear bond strength (*P* < 0.05) to composite than the groups that utilized bonding agents. *Conclusion*. Composites bonding to stainless steel crowns using the new universal bonding agent (Scotchbond Universal Adhesive, 3M ESPE, Seefeld, Germany) show significantly greater shear bond strengths and fewer adhesive failures when compared to traditional single-bottle systems.

## 1. Introduction 

Stainless steel crowns (SSCs) are most commonly used for full coverage restoration of posterior primary teeth [1–3]. Given the nature of primary enamel, the need for a full coverage restoration in children is all the more important [4]. For children who presented with large, multisurface carious lesions of the primary teeth, the American Academy of Pediatric Dentistry recommended the full coverage of crowns using SSCs [5]. Despite their high success rate, this proven restoration often fails to meet the esthetic demands of patients and their parents [3, 6].

Esthetic SSCs are composite or porcelain coatings that are chemically or mechanically attached to a metal coping which allows for a tradeoff between their respective strengths and weaknesses [6]. Esthetic SSCs have several shortcomings relative to traditional SSC restorations such as requiring a greater reduction of tooth structure during preparation [7], inability to crimp the crown [8], and repair of fractured coatings sometimes requiring complete replacement [9]. The shape of an esthetic SSC cannot be altered, because this would change the rigid metal coping structure beneath the somewhat brittle composite, leading to the possibility of future fracture of the composite [10]. Although there is documentation of the repair of fractured esthetic crowns [11], replacement of the crown is often the only method of managing such failures [9]. 

A treatment modality that allows for contouring of the crown as well as adequate retention is the chair-side veneering of composite to stainless steel crowns [12]. The use of such crowns has been restricted by the poor esthetics of the metal display due to inadequate bonding of the metal to composite [13].

Recently, a dental adhesive has been developed for multipurpose bonding (Scotchbond Universal, 3M-ESPE, Seefeld, Germany). This adhesive, developed for repairs in prosthodontic crowns, utilizes self-etch phosphorylated methacrylates that are believed to result in more efficacious bonding to metal as well as tooth structure [14, 15]. However, to the researchers knowledge this system has not been tested on stainless steel crowns. Given the better bonding of nanocomposites to stainless steel crowns than conventional composites [16], this study aimed to evaluate the bonding of nanocomposites to stainless steel crowns using a universal bonding system.

## 2. Materials and Methods

### 2.1. Selection of Materials

This study tested the bond strength of commercially available nanocomposite resins to pretrimmed, precontoured posterior stainless steel crowns, (3M, St. Paul, MN, USA) using a new universal bonding system Scotchbon (Universal Adhesive, 3M ESPE, Seefeld, Germany). The bond strength obtained was compared to that of using two currently available dentin bonding agents Adaper Single Bond Plus (3M ESPE, Seefeld Germany) and Prime & Bond NT (Dentsply, Lichtenstein).

To avoid any chance of incompatibility of the bonding agent with the composite, each bonding agent was tested with a resin manufactured by the same manufacturer (ZX350, 3M ESPE; Ceram-X mono, Dentsply).

### 2.2. Power of the Sample

The power of the sample was calculated using the G-Power 3.1.3 power analysis software (Universtät Kiel, Germany). The minimum required sample for the one-way ANOVA and post-hoc test, with alpha of 0.05, was 20 samples in each group. Thus a total of 80 stainless steel crowns were divided into four groups for the purpose of this study.

### 2.3. Preparation of the Stainless Steel Crowns

The lingual surfaces of 80 pretrimmed, precontoured lower right primary secondary molar crowns (size E5) (3M, St Paul) were sandblasted for 20 seconds using a sandblasting machine to increase retention (Protempomatic Z, Bego, Bremen, Germany). Then the crowns were etched with 37% phosphoric acid (FineEtch 37, Spident Co. Ltd., Korea) for 15 seconds and divided into four groups of 20 SSCs each. In Group A SSCs were attached to a nanocomposite (ZX350, 3M-ESPE) without the use of a bonding agent (control group). In Group B SSCs were bonded to a nanocomposite (ZX350, 3M-ESPE) using the universal bonding system (Scotchbond Universal Adhesive, 3M ESPE). In Groups C and D SSCs were bonded to nanocomposites using bonding agents from their respective manufacturers (Adaper Single Bond Plus to ZX350, 3M ESPE, and Prime & Bond NT to Ceram-X Dentsply). After that all SSCs were subjected to thermocycling (500 cycles of thermocycling between 5°C and 55°C) to simulate thermal changes in the oral cavity using the technique. 

### 2.4. Measurement of Bond Strength

**Figure 1:**
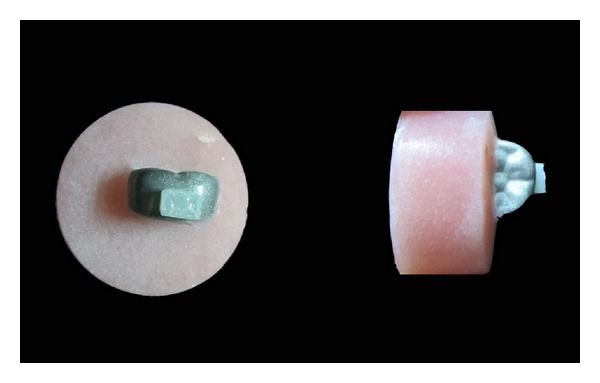
Stainless steel crown bonded to composite, mounted in an acrylic resin template.

In order to facilitate handling of the samples the crowns were embedded in a uniform acrylic mold that exposed the lingual surface of each crown to be ready for testing (Figure 1). Then the shear bond strength of the composite to the stainless steel crown was measured using a universal testing machine (Instron Corp, Canton, MA, USA). A force of 10 N at an acceleration of 0.5 mm/min was applied upon the crown-composite interface in a direction parallel to the long axis of the crown. All strength readings were calculated in megapascals (MPa) and the force at which the bond fractured was recorded as the shear bond strength of the adhesive.

### 2.5. Types of Failure

**Figure 2:**
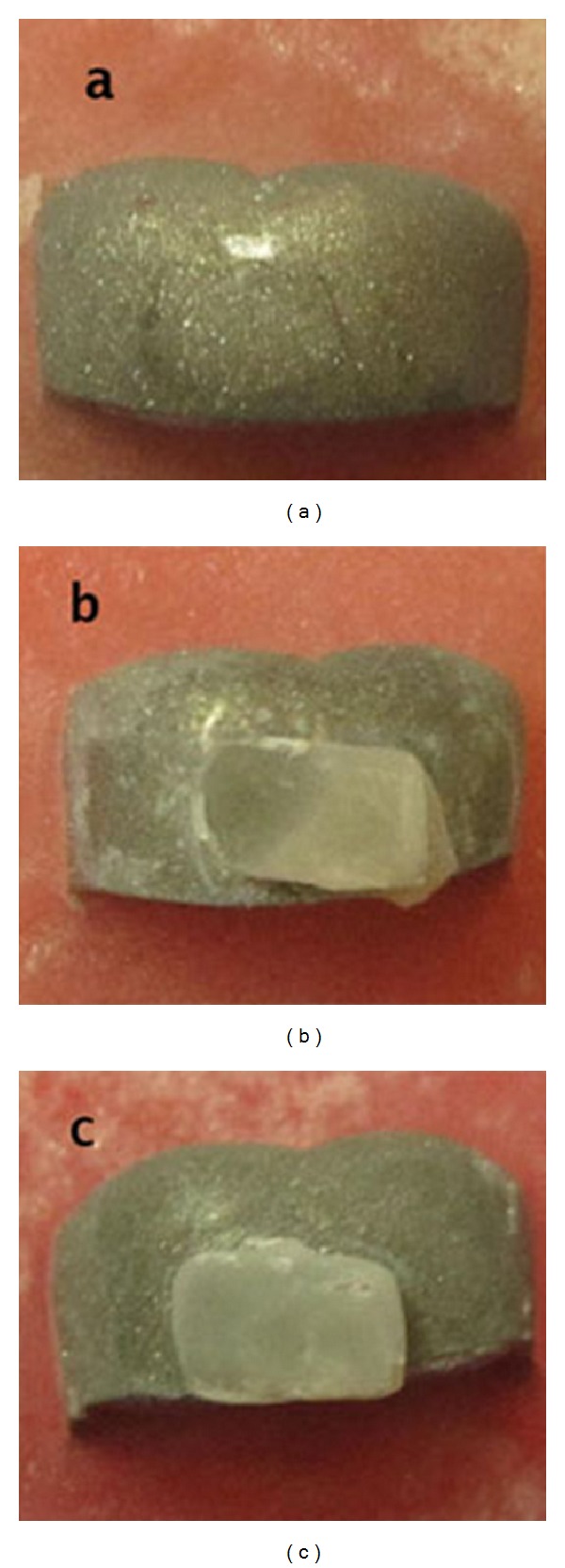
Types of failure of shear bond strength: (a) adhesive failure, (b) mixed failure, and (c) cohesive failure.

After the fracture of the bond between the composite and the stainless steel crown, the crown samples were examined under a magnifying loupe and a dark background to determine type of failure (Figure 2). Three distinct types of failure were recorded: (a) adhesive failure was recorded when the bond failure was observed at the resin-stainless steel crown interface, (b) cohesive failure was recorded when the bond failure was observed within the resin and (c) mixed failure when the bond failure was located at the resin stainless steel crown as well as within the resin [15].

### 2.6. Statistical Analyses

 All data was recorded and processed using the SPSS ver.20 (IBM Inc., Armonk, NY, USA) data processing software. The one-way ANOVA was used to compare the significance of difference in shear bond strengths between the different groups. Intergroup variations were further illustrated using Scheffe's post-hoc test. Pearson's Chi Square was used to determine the significance of difference among the types of failure.

## 3. Results

Table 1 shows the mean, standard deviations and coefficients of variance of the shear bond strengths of the different groups tested and subjected to the one-way ANOVA. The control group (without any bonding agent) had significantly lower shear bond strength to composite than the groups that utilized bonding agents. The one-way ANOVA showed that this difference was significant at *P* < 0.0001. Post-hoc Scheffe's test (Table 2) suggested that the shear bond strength of Group B (Scotchbond Universal Adhesive) was significantly higher than that of both Group C (Prime & Bond NT) and Group D (Adaper Single bond Plus). No significant differences were found between the shear bond strengths of Group C and Group D (*P* = .897).

When the failure type of bonding was compared, the control group showed only adhesive failure, while groups C and D showed both adhesive and mixed failures. Although the universal bonding agent group (Group B) showed two samples with pure cohesive failure, this group had an equal number of adhesive and mixed failures (Table 3). The type of failure observed in this group was however significantly different from those observed in other groups.

## 4. Discussion

Full coverage restorations are often the only viable means of restoring badly decayed primary teeth [17–19]. Pediatric dentists have recognized the need for an esthetic alternative to stainless steel crowns for anterior teeth [17], and similarly posterior teeth too; parental demand for esthetics has forced pediatric dentists to look at more esthetic options such as preveneered or open-faced crowns [19]. 

Although parent satisfaction has been reported with pre-veneered stainless steel crowns [20], drawbacks such as difficulties in shade matching [17], tendencies for the veneered surfaces to fracture [9], limited ability to crimp the crown [21], and fears over long-term clinical performance [22] have prevented their universal acceptance by dentists. A higher rate of clinical success has been reported with open-faced stainless steel crowns although their esthetic acceptability has been questioned [23].

While initial studies on the bonding of composite to stainless steel crowns predicted acceptable shear bond strengths [12, 24, 25], it has been found that mechanical modifications improve the bond strength [26, 27]. Sandblasting was chosen as the method to improve retention of the composite to the crown as the equipment is available in most labs and since it is less time consuming and easier to standardize when compared to methods such as welding of orthodontic brackets or creation of grooves manually with a bur [26]. 

The shear bond strength obtained with the single bottle adhesives were in the range obtained by previous studies using similar adhesives [16, 27]. The lack of any significant difference between manufacturers seems to validate the initial hypothesis and confirm the need to test newer bonding systems. The results showed that the universal bonding system showed a significantly higher shear bond strength and lower incidence of adhesive failure than both single bottle adhesives indicating that there could be potential for clinical applications of this system in bonding composite to stainless steel.

The bonding failure type has been used as a measure of the success of the bond of adhesive restorations to stainless steel crowns [16, 27]. Adhesive failures have been considered unacceptable, mixed failures acceptable, and cohesive failures ideal [15]. While studies have reported adhesive and mixed failures in the bonding of composite to stainless steel crowns there have been few reports of cohesive failure [15]. In this context, the significantly fewer adhesive failures and finding of cohesive failure in the group that bonded with the universal bonding agent are of significance. However it must be noted that even the group that bonded with universal bonding agent showed incidence of adhesive failure, suggesting that clinical trials of this material are needed to validate the findings of this study.

Because of the relative novelty of the universal bonding agent, there is little available literature on the mechanism of action of this agent. A recent study suggested using self-etching adhesives that utilize 10-methacryloyloxydecyl dihydrogen phosphate (MDP) form self-assembled nanolayers at the tooth-bond interface, which could be the reason for their higher bond strengths to tooth [15]. However both the presence of this layer and the feasibility of this explanation with regards to metal bonding need further evaluation. This study used only the universal bonding agent since none of the other commercially available MDP systems claim increased bonding to metal.

The fracture of the veneering of pre-veneered stainless steel crowns has been reported to result in loss of space and retention of plaque [28, 29]. The repair of the fractured veneer has been reported to be unsuccessful, mainly because of the lack of adequate bonding to the metal surface [28]. The results of this study suggest that the higher bond strengths of universal bonding agent could indicate the need to study their possible use in the repair of fractured veneers of esthetic stainless steel crowns.

## 5. Conclusions

Composites that bonded to stainless steel crowns with the new universal bonding agent show significantly greater shear bond strengths and fewer adhesive failures when compared to traditional single bottle systems. Further clinical research is needed to evaluate the in vivo potential of this system.

## Figures and Tables

**Table 1:** Descriptive data of the shear bond strengths of the different groups.

Group	*N*	Mean	Std. Deviation	Std. Error	*F*	Sig*
Spectrum + Prime & Bond NT (D)	20	9.7700	5.40089	1.20768	32.887	0.000**
Z350XT + Adaper (C)	20	10.7460	5.09795	1.13994		
Z350XT + Universal (B)	20	17.6200	4.21568	.94265		
Control (A)	20	3.7950	2.26053	.50547		

*Calculated using one-way ANOVA.

**Differences significant at *P* < 0.001.

**Table 2:** Tukey's HSD post-hoc test to highlight significant differences between the different groups.

Group	*N*	Subset for alpha = 0.05		
		1	2	3
Control (A)	20	3.7950		
Spectrum + Prime & Bond NT (D)	20		9.7700	
Z350XT + Adaper (C)	20		10.7460	
Z350XT + Universal (B)	20			17.6200
Sig.		1.000	.897	1.000

Means for groups in homogeneous subsets are displayed.

a. Uses harmonic mean sample size = 20.000.

**Table 3:** Types of failure observed.

Group	Type of failure			Chi Square	Sig
	Adhesive	Cohesive	Mixed
Spectrum + Prime & Bond NT	16	0	4		
Z350XT + Adaper	14	0	6	9.254	0.004*
Z350XT + Universal	9	2	9		
Control	20	0	0		

*Differences significant at *P* < 0.01.
